# Disruption of the Anterior Borders of the Mammary Gland on Ultrasound Does Not Always Indicate the Presence of Invasive Breast Cancer Components: A Case Report

**DOI:** 10.7759/cureus.111248

**Published:** 2026-06-21

**Authors:** Aoi Suenaga, Shoji Oura

**Affiliations:** 1 Department of Surgery, Kishiwada Tokushukai Hospital, Kishiwada, JPN

**Keywords:** back ground fibrous stroma, breast cancer, disruption of anterior borders, mammary gland, non-invasive cancer

## Abstract

Disruption of the anterior borders of the mammary gland on ultrasound usually indicates the presence of invasive breast cancer components in the tumor. A 60-year-old woman was referred to our hospital for a detailed examination of an abnormality on screening mammography. Mammography showed distortion of the mammary gland and no definite masses in the left breast. Ultrasound also did not depict any masses but showed low echoes with indistinct margins and disruption of the anterior borders of the mammary gland. MRI of the target lesion showed low signals on T1-weighted images and very weak high signals on fat-suppressed T2-weighted images, with initial slow enhancement followed by a plateau pattern on dynamic studies. These imaging findings led us to perform a vacuum-assisted biopsy of the target areas, which pathologically showed non-invasive cancer cells growing in a cribriform fashion.

Despite the lack of invasive components in the biopsy specimen, the patient underwent breast-conserving surgery and sentinel node biopsy to avoid undertreatment. Frozen section confirmed no lymph node metastasis and negative surgical margins. Postoperative pathological examination showed that the breast cancer had sparse ductal carcinoma in situ growing in cribriform and solid patterns, along with focal depression of the background fibrous stroma edges at the presumed disruption areas of the mammary gland anterior borders. The patient recovered uneventfully, has received adjuvant radiotherapy to the conserved breast, and has been well on endocrine therapy for four months. Diagnostic physicians should note that disruption of the anterior borders of the mammary gland on ultrasound does not always indicate the presence of invasive breast cancer components.

## Introduction

Mammography can provide objective images of breast disorders and therefore plays the most important role in the diagnosis of breast cancer [1]. For example, spiculated masses or fine linear calcifications on mammography directly lead to a Breast Imaging Reporting and Data System Category 5 diagnosis [2]. In addition, screening mammography can reduce breast cancer mortality and has been the mainstay of breast cancer screening programs. On the other hand, mammography sometimes cannot depict even large tumors when they are located in dense breasts [3].

Ultrasound has certain drawbacks, including its inability to visualize the entire breast and its operator-dependent nature, leading to poor objectivity. However, ultrasound can provide cross-sectional images and easily depict small masses even in dense breasts [4]. Ultrasound can furthermore provide various useful information about tumor aggressiveness in addition to the location, size, and shape of breast cancer. For instance, the presence of distinct lymph node metastasis and skin thickening on ultrasound can suggest that the breast cancer has aggressive characteristics with high lymphatic permeability [5]. In addition, disruption of the anterior borders of the mammary gland generally leads us to suspect invasive breast cancer [6].

When breast surgeons suspect possible lymph node metastasis through palpation of small but hard lymph nodes or detection of partial thickening of the lymph node cortex on imaging, they often perform a sentinel lymph node biopsy even if the pathological results of a needle biopsy indicate ductal carcinoma in situ (DCIS), to avoid under-treatment. We report a case of grade 1 DCIS in which the presumed disruption of the anterior borders of the mammary gland on ultrasound led to a preoperative misinterpretation of the lesion as having invasive breast cancer components.

## Case presentation

A 60-year-old woman with a history of ossification of the posterior longitudinal ligament was referred to our hospital for detailed evaluation of an abnormal finding on screening mammography. The patient had no family history of breast or ovarian cancer, but had a family history of hepatocellular carcinoma in her father. Mammography only showed distortion of the mammary gland in the left breast (Figure 1).

**Figure 1:**
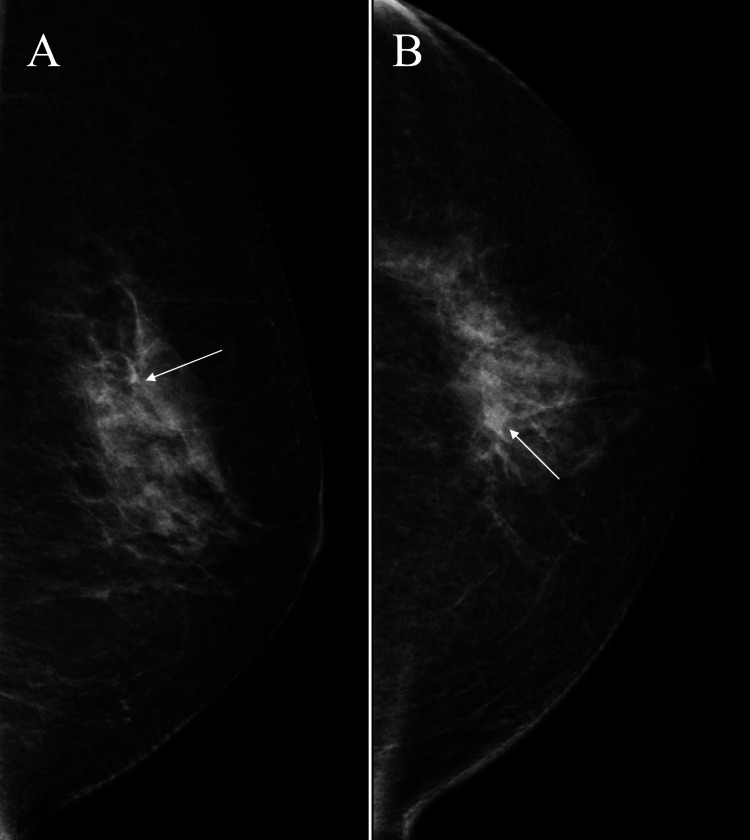
Mammography findings Mammography showed distortion of the mammary gland (arrow) and no masses, both on the mediolateral oblique view (A) and the craniocaudal view (B)

Ultrasound also did not depict any masses in the left breast, but showed low echoes with indistinct margins and the disruption of the anterior borders of the mammary gland (Figure 2).

**Figure 2:**
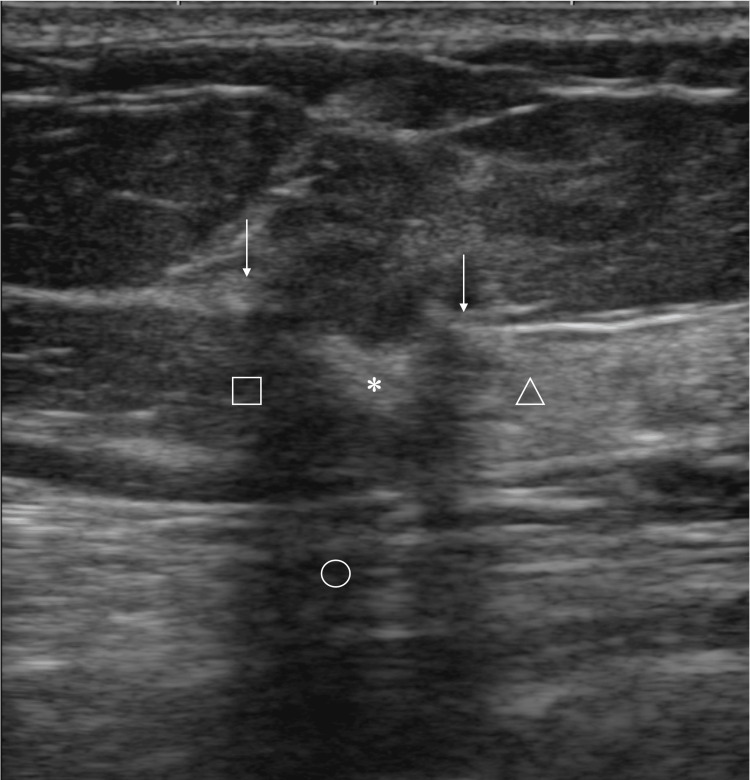
Ultrasound findings Ultrasound showed disruption of the mammary gland anterior borders (arrows), high echoes in the non-mass forming lesion (asterisk), and slight attenuation of posterior echoes (open circle). Ultrasound further showed high echoes suggesting edematous stroma just under the anterior borders of the mammary gland on one side (open triangle), and not on the other side (open square)

MRI of the target lesion showed low signals on T1-weighted images, very weak high signals on fat-suppressed T2-weighted images, and initial slow enhancement followed by a plateau pattern on dynamic studies (Figure 3).

**Figure 3:**
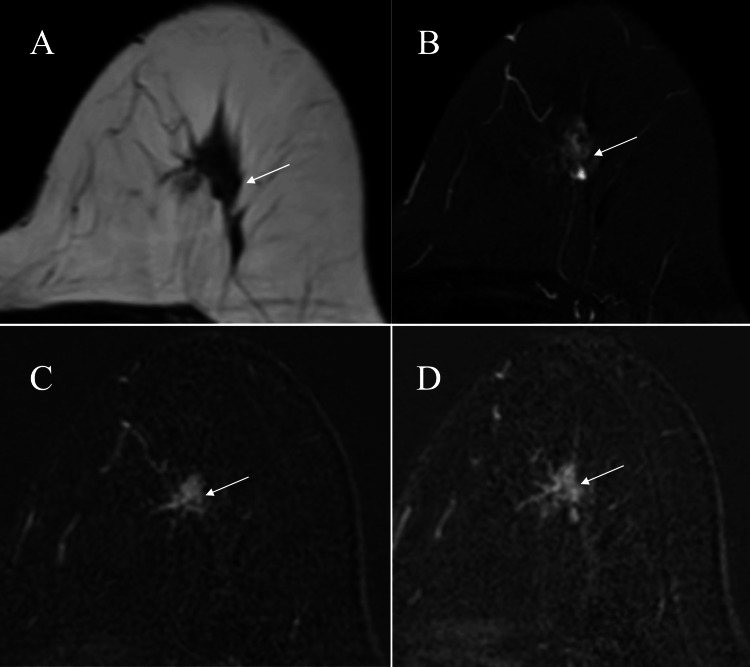
MRI findings MRI of the target lesion (arrow) showed low signals on T1-weighted images (A), very weak high signals on fat-suppressed T2-weighted images (B), and slow early enhancement (C) and retained enhancement (D) on dynamic studies MRI: magnetic resonance imaging

The patient, therefore, underwent vacuum-assisted biopsy (VAB) of the target lesion under the tentative diagnosis of cancer cell-sparse invasive breast cancer. VAB pathologically showed non-invasive cancer cells growing in a cribriform fashion. Despite the lack of invasive components in the VAB specimen, the patient underwent breast-conserving surgery and sentinel node biopsy to avoid undertreatment. Frozen section clarified no lymph node metastasis and negative surgical margins. Postoperative pathological study showed that the resected specimen had sparse grade 1 DCIS foci growing in cribriform and solid fashions, sclerosing adenosis at the anterior borders of the background fibrous stroma, negative surgical margins, i.e., 5 mm to the lateral borders, and focal depression of background fibrous stroma edges at the presumed disruption areas of the mammary gland anterior borders (Figure 4).

**Figure 4:**
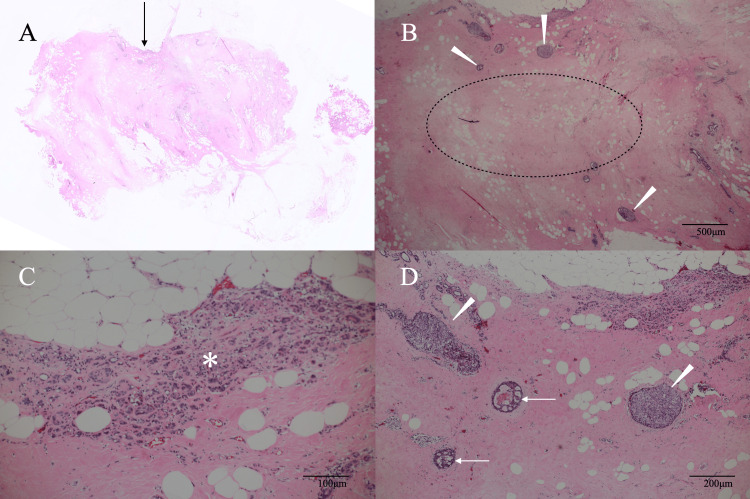
Pathological findings (H&E staining) A. Low magnified view showed the depression (arrow) of the background fibrous stroma edges at the disruption areas of the mammary gland anterior borders on ultrasound (H&E ×4). B. Magnified view showed sparsely distributed DCIS foci (arrowheads) and many fat cells (arrows) under the depressed areas of the background fibrous stroma edges (H&E ×40). C. Magnified view showed that sclerosing adenosis (asterisk) located at the anterior edges of the fibrous background and faced the fat tissue (H&E ×200). D. Magnified view showed that cribriform (arrows) and solid (arrowheads) type grade 1 DCIS foci sparsely located in the fibrous background (H&E ×100) H&E: hematoxylin and eosin; DCIS: ductal carcinoma in situ

Immunostaining showed that cancer cells were positive both for estrogen and progesterone receptors (both Allred score 5), human epidermal growth factor receptor type 2 equivocality (fluorescence in situ hybridization negative), and a Ki-67 labelling index of 16% (Figure 5).

**Figure 5:**
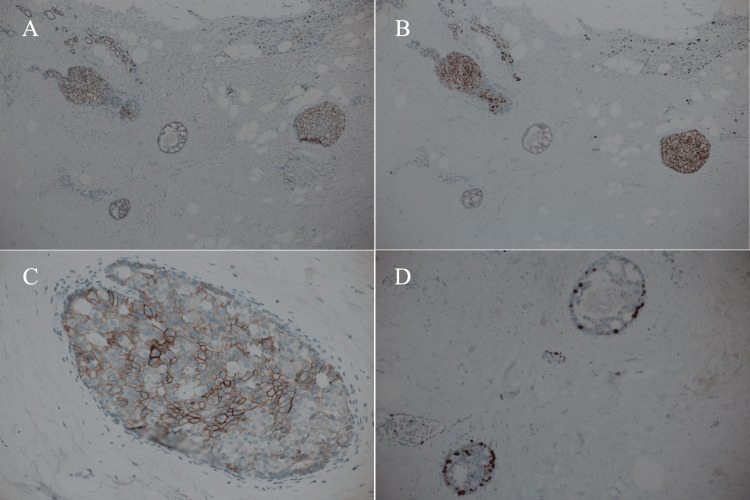
Pathological findings (immunostaining) Immunostaining showed estrogen (A) and progesterone (B) receptor positivities (both Allred score 5), human epidermal growth factor receptor equivocality (C), and a Ki-67 labelling index of 16% (D)

The patient recovered uneventfully. She was discharged on the second day after surgery, received adjuvant radiotherapy to the conserved breast, has been well for four months on endocrine therapy, and is scheduled for long-term outpatient follow-up.

## Discussion

It is well known that distortion of the mammary gland closely correlates with the presence of fibrous components [7]. Therefore, scirrhous-type invasive ductal carcinoma and invasive lobular carcinoma, both having abundant fibrous components, often show distortion of the mammary gland. DCIS in the present case demonstrated distortion on mammography and pathologically had sparse cancer cells within abundant fibrous components, corresponding well with the imaging findings such as attenuated posterior echoes, very weak high signals on fat-suppressed T2-weighted images, and initial slow enhancement followed by persistent enhancement on dynamic studies [8,9].

It has already been clarified that the normal mammary gland has parenchyma formed by terminal duct lobular units (TDLUs) and perilobular stroma. The perilobular stroma consists of two components: the surrounding stroma, which surrounds the TDLUs, and the edematous stroma, which occupies the space between TDLUs with the surrounding stroma and often contains fat cells. Ultrasound shows the former as hypoechoic and the latter as hyperechoic [10]. In addition, aging does not affect the former but reduces the latter and replaces some parts of it with fat cells. We judged that the anterior borders of the mammary gland were disrupted based only on the lack of continuity of these borders.

We, however, did not take the absence of high echoes of the mammary gland, suggesting edematous stroma, into account when judging the clinical implications of the anterior border disruption of the mammary gland, leading to the overestimation of the ultrasound findings. In addition, high echoes just beneath the disrupted areas of the anterior borders in the non-mass-forming lesion further led us to over-evaluate the status of the mammary gland anterior border. Pathological findings, however, clarified that ultrasound backscattering due to the intermingling of numerous fat cells within the background fibrous stroma generated these high echoes in the non-mass-forming lesion.

DCIS components were located very sparsely within the fibrous background in this case. The areas that we interpreted as anterior border disruption of the mammary gland showed focal irregular depressions of the background fibrous stroma edges. These irregular depressions had different angles from those of the adjacent fibrous stroma edges, leading to little or no ultrasound wave reflection back toward the ultrasound probe. These pathological characteristics generated the apparent disruption of the anterior mammary gland border on ultrasound.

It naturally remains uncertain why these background fibrous stroma edges are irregularly depressed. In addition, we cannot even speculate whether the sclerosing adenosis is correlated with the focal depression of the background fibrous stroma edges. In any case, diagnostic physicians should note that disruption of the mammary gland anterior borders, when lacking high echoes due to the presence of edematous stroma just beneath the disruption areas, does not always imply the presence of invasive components and can lead to overestimation of the ultrasound findings.

## Conclusions

Disruption of the anterior borders of the mammary gland on ultrasound generally suggests the presence of invasive cancer components and is an important finding predictive of the pathological characteristics of breast cancer. When ultrasound shows anterior border disruption of the mammary gland, breast surgeons generally perform sentinel lymph node biopsy to avoid undertreatment, even if invasive components are absent in the needle biopsy specimen. Physicians, however, should note that focal depression of background fibrous stroma edges can generate the appearance of mammary gland anterior border disruption even in DCIS. In addition, breast surgeons should note that disruption of the anterior borders of the mammary gland on ultrasound does not always indicate the presence of invasive components.
